# Circadian and Brain State Modulation of Network Hyperexcitability in Alzheimer’s Disease

**DOI:** 10.1523/ENEURO.0426-17.2018

**Published:** 2018-05-17

**Authors:** Rosalind Brown, Alice D. Lam, Alfredo Gonzalez-Sulser, Andrew Ying, Mary Jones, Robert Chang-Chih Chou, Makis Tzioras, Crispin Y. Jordan, Izabela Jedrasiak-Cape, Anne-Laure Hemonnot, Maurice Abou Jaoude, Andrew J. Cole, Sydney S. Cash, Takashi Saito, Takaomi Saido, Richard R. Ribchester, Kevan Hashemi, Iris Oren

**Affiliations:** 1 Centre for Discovery Brain Sciences, University of Edinburgh, Edinburgh, EH8 9JZ, United Kingdom; 2 Epilepsy Division, Dept of Neurology, Massachusetts General Hospital, Boston, MA 02214; 3 Harvard Medical School, Boston, MA 02214; 4 Université de Montpellier, Montpellier, 34000 France; 5 Laboratory for Proteolytic Neuroscience, RIKEN Center for Brain Science, Saitama, 351-0198 Japan; 6 OpenSource Instruments Inc, Watertown, MA 02472

**Keywords:** Alzheimer’s disease, circadian cycle, epilepsy

## Abstract

Network hyperexcitability is a feature of Alzheimer’ disease (AD) as well as numerous transgenic mouse models of AD. While hyperexcitability in AD patients and AD animal models share certain features, the mechanistic overlap remains to be established. We aimed to identify features of network hyperexcitability in AD models that can be related to epileptiform activity signatures in AD patients. We studied network hyperexcitability in mice expressing amyloid precursor protein (APP) with mutations that cause familial AD, and compared a transgenic model that overexpresses human APP (hAPP) (J20), to a knock-in model expressing APP at physiological levels (APP^NL/F^). We recorded continuous long-term electrocorticogram (ECoG) activity from mice, and studied modulation by circadian cycle, behavioral, and brain state. We report that while J20s exhibit frequent interictal spikes (IISs), APP^NL/F^ mice do not. In J20 mice, IISs were most prevalent during daylight hours and the circadian modulation was associated with sleep. Further analysis of brain state revealed that IIS in J20s are associated with features of rapid eye movement (REM) sleep. We found no evidence of cholinergic changes that may contribute to IIS-circadian coupling in J20s. In contrast to J20s, intracranial recordings capturing IIS in AD patients demonstrated frequent IIS in non-REM (NREM) sleep. The salient differences in sleep-stage coupling of IIS in APP overexpressing mice and AD patients suggests that different mechanisms may underlie network hyperexcitability in mice and humans. We posit that sleep-stage coupling of IIS should be an important consideration in identifying mouse AD models that most closely recapitulate network hyperexcitability in human AD.

## Significance Statement

It is increasingly recognized that Alzheimer’s disease (AD) is associated with hyperexcitability in brain networks. Brain network hyperexcitability is also reported in several rodent models of AD. We studied the signatures of this hyperexcitability in two rodent models of AD as well as AD patients. Network hyperexcitability was prevalent in a transgenic model of AD but was absent in a rodent model that is considered to be more physiologic. Moreover, while network hyperexcitability was coupled to rapid eye movement (REM) sleep in transgenic mice, hyperexcitability occurred in non-REM (NREM) sleep in AD patients. We suggest that brain state coupling of hyperexcitability can be used as a method for screening animal models of AD.

## Introduction

An increased incidence of seizures in Alzheimer’s disease (AD) is indicative of an underlying network hyperexcitability (Hesdorffer et al., 1996; Amatniek et al., 2006; Lozsadi and Larner, 2006; Vossel et al., 2013; Cretin et al., 2016). Interictal spikes (IIS) are also seen in a high proportion of AD patients without a history of clinical seizures (Vossel et al., 2016). Nonictal network hyperactivity has also been detected by means of fMRI in individuals at risk of developing dementia, for example in people carrying the APOE4 allele (Bookheimer et al., 2000; Filippini et al., 2009), with other genetic predictors of AD (Quiroz et al., 2010) and also in patients with mild cognitive impairment (MCI), a diagnosis which is considered to be a prodromal stage of AD (Dickerson et al., 2005). Network hyperexcitability and seizure activity appear at early stages of the disease and have been suggested to be predictors of accelerated cognitive decline (Amatniek et al., 2006; Vossel et al., 2013; Cretin et al., 2016; Vossel et al., 2016).

Network hyperexcitability has also been reported in numerous mouse models of AD pathology (Palop et al., 2007; Minkeviciene et al., 2009; Busche et al., 2012; Šišková et al., 2014; Kazim et al., 2017), with the aberrant activity being a feature that occurs in advance of plaque deposition (Busche et al., 2012; Bezzina et al., 2015). These phenomenological similarities have led to the suggestion that these animal models can provide a tool by which to study network hyperexcitability in human AD (Palop and Mucke, 2016).

Aberrant network activity could in itself contribute to neurodegeneration and cognitive dysfunction in AD pathology (Cirrito et al., 2005; Bero et al., 2011; Busche and Konnerth, 2015; Wu et al., 2016). Reducing network hyperexcitability has been shown to ameliorate cognitive dysfunction in both patients and animal models (Bakker et al., 2012; Sanchez et al., 2012; Haberman et al., 2017), and to attenuate Aβ pathology (Yuan and Grutzendler, 2016). Hence, targeting network hyperexcitability has been suggested as a novel therapeutic avenue for AD. However, studying this therapeutic avenue by means of animal models (Sanchez et al., 2012) requires a deeper understanding of the shared features of network hyperexcitability between AD patients and animal models.

Expression of epileptiform activity frequently exhibits a circadian pattern and shows preferential activation with specific brain states in a range of epilepsies (Quigg, 2000; Ng and Pavlova, 2013; Sedigh-Sarvestani et al., 2015). Circadian dysfunction and sleep disruption are common features of AD and are also considered as early features of disease pathogenesis (Musiek et al., 2015; Mander et al., 2016; Musiek et al., 2018). Two recent papers have reported modulation of epileptiform activity by circadian cycle and brain state in transgenic AD models. Epileptiform activity was more prevalent in daylight conditions, and was suggested to occur primarily during rapid eye movement (REM) sleep (Born et al., 2014; Kam et al., 2016). If epileptiform activity is modulated by circadian cycles and/or brain state in AD patients, it is possible that this might contribute to the reported circadian alterations and sleep dysfunction. In line with this, it has recently been shown that interictal activity in AD patients is highly prevalent during sleep (Vossel et al., 2016; Horváth et al., 2017; Lam et al., 2017). The modulation of ictal related activity by brain state points to a distinguishing feature that could be used to (1) uncover distinct mechanisms underlying hyperexcitability, and (2) ascertain the translational utility of specific animal models in studying network hyperexcitability. To this end, the present study aimed to investigate circadian and brain state modulation of network hyperexcitability in two rodent models of AD of differing etiology: one in which human amyloid precursor protein (hAPP) is overexpressed and one in which APP is expressed at endogenous levels. In order to shed light on the translational utility of rodent AD models for studying network hyperexcitability in human AD, we further examined sleep-stage modulation of epileptiform activity in two patients with AD, using recordings from intracranial electrodes placed directly adjacent to the hippocampus.

## Materials and Methods

### Animals and animal maintenance

All animal procedures were performed in accordance with the University of Edinburgh animal welfare committee regulations and were performed under a United Kingdom Home Office project license.

Heterozygous mice (+/−) expressing hAPP with the KM670/671NL (Swedish) and V717F (Indiana) mutations on a PDGFβ promoter (J20; Mucke et al., 2000) were bred by crossing J20 +/− (i.e. animals are heterozygous) males with C57Bl6J females. Experiments used J20 +/− (*n* = 21) and J20-/- (*n* = 8) wild-type (WT) littermate controls. The mean age of J20 animals was five months (range: 3.3–6.5 months).

Homozygous knock-in mice expressing APP KM670/671NL (Swedish) and APP I716F (Iberian) mutations (APP^NL/F^; Saito et al., 2014) were back-crossed onto C57Bl6J strain for at least three generations and were >99.8% cogenic with C57Bl6J. Experiments used APP^NL/F^ +/+ (*n* = 20) and age-matched nonlittermate C57Bl6J WT controls (*n* = 15). Animals were either eight or 12 months of age.

Both male and female mice were used. Mice were kept on a 7/19 h light/dark cycle in standard, open cages. Mice were group-housed before surgery and were housed individually postsurgery and during telemetry data acquisition.

### Surgery and data acquisition

A subdural intracranial electrocorticogram (ECoG) recording electrode was positioned in the cortex overlying the hippocampus (coordinates *x*: −2.25; *y*: −2.46). A reference electrode was implanted either in the skull of the contralateral hemisphere, or above the cerebellum. Electrodes were either bare wire, or skull screws. An EEG transmitter (A3028B, Open Source Instruments) was implanted on the back of the animal subcutaneously. Animals were left to recover for at least 24 h after surgery before the commencement of telemetry data acquisition. Telemetric ECoG data were acquired for ∼3 d from each animal. Recording was either conducted continuously between days 1 and day 3 after surgery, or day 1, followed by day 5 to day 6.

ECoG data were acquired using an Opensource Instruments data acquisition system at 512 sps as previously described (Chang et al., 2011).

Video data were acquired using a Basler acA1300-60gm GigE camera sampling at 10 fps, or a Logitech C270 HD webcam sampling at 5 fps. Video was acquired during the daylight hours.

### ECoG data processing

The raw ECoG data were analyzed using custom written Tcl and C processors. ECoG data were analyzed in 8-s intervals. For each interval we extracted measures of data loss, spike count, δ power (0.1–3.9 Hz) and θ power (4–12 Hz). We defined intervals in which data loss exceeded 20% of samples as “lossy” intervals. Intervals in which δ power exceeded 0.16 mV^2^ were classified as artifacts. Lossy and artifact intervals were excluded.

IIS in rodent ECoG were detected as follows. Each 8-s interval of EEG was treated as a two-dimensional path. One dimension is voltage, which was normalized by dividing by the mean absolute step size of the voltage in the 8-s interval. The mean absolute step size is the sum of the absolute changes in voltage from one sample to the next, divided by the number of samples. For an 8-s interval, the number of samples would be 4096 and a typical mean absolute step size for mouse EEG is around 12 µV. The other dimension is time, which was normalized by dividing by the sample period. The spike-finder proceeds along this EEG path in steps. With each step, it moves to the nearest sample on the path ahead. Whenever the spike-finder steps past one or more samples, it classifies these samples as an aberration in the path. Solitary aberrations larger than 20 mean absolute step sizes are classified as IIS. A series of IIS in which single spikes were separated by <78 ms (40 samples) were treated as a burst event and counted as one IIS event within the 8-s interval.

For each J20 animal, the false positive rate of IIS detection was determined by randomly hopping through 100 8-s intervals identified by the processor as containing IIS and scoring them as true or false positives. The animal was excluded from analysis if the false positive rate exceeded 10%. One animal was excluded from analysis on this basis. In the remaining animals, the false positive rate ranged from 0% to 6% (mean false positive rate: 1.9%).

**Figure 1.**
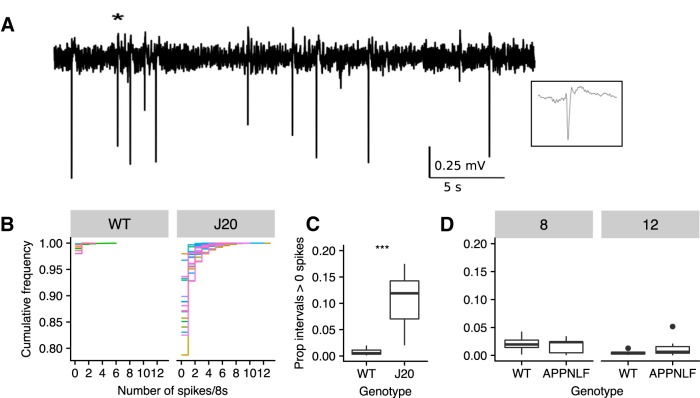
IISs are prevalent in J20 mice but not in APP knock-in mice. ***A***, ECoG trace recorded from a J20 mouse showing IIS. Inset is 250-ms expansion around IIS event marked by *. ***B***, Empirical cumulative distribution frequency plots for individual animals quantifying the number of detected IIS in 8-s intervals across 3 d of recording in WT and J20s. Colors represent distributions for individual animals. ***C***, Plot showing the proportion of intervals with one or more detected IIS in WT and J20. ***D***, Plot showing the proportion of intervals with one or more detected IIS in WT and APP^NL/F^ at eight and 12 months. Bars represent medians. Whiskers extend to 1.5 interquartile range and data points outside of this range shown as points; ***p < 0.001.

**Figure 2.**
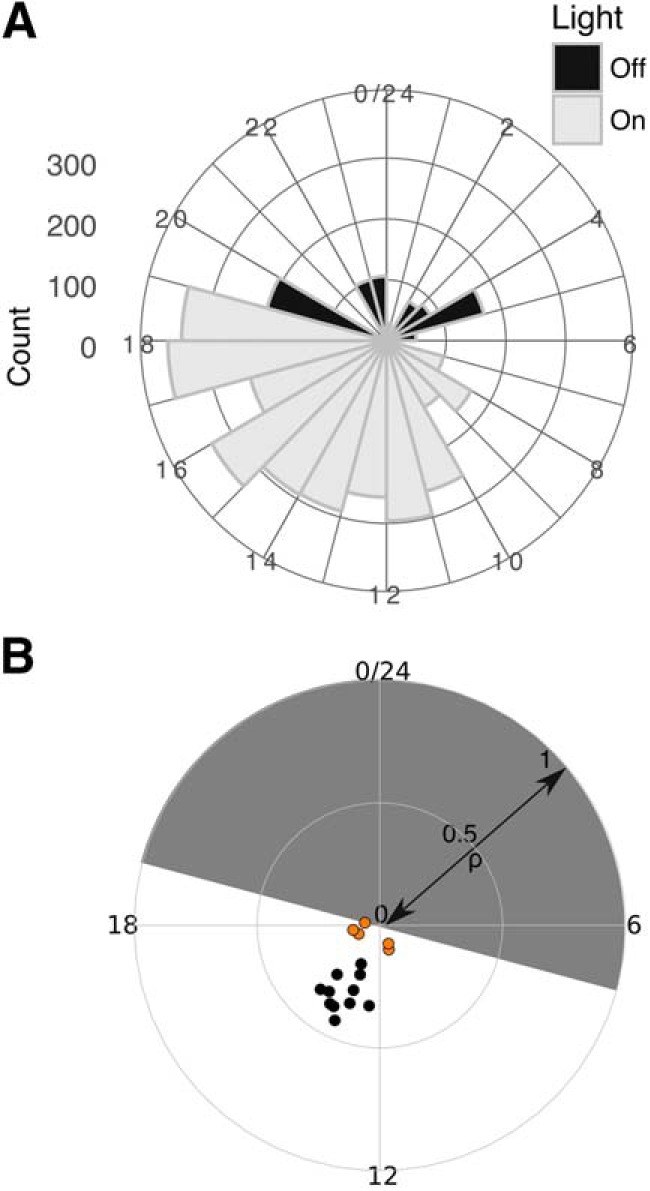
Circadian modulation of IIS. ***A***, Circular histogram of IIS counts over 3 d of recording in an individual J20 mouse plotted on 24-h cycle. Light condition indicated by shading. For the animal shown, $\varphi_{IIS}$ = 14 h 51 min and ρ = 0.35. ***B***, Summary data for $\varphi_{IIS}$ versus ρ for all animals, shown on circular plot. Solid symbols are strongly-coupled animals. Weakly coupled animals are shown with orange fill.

**Figure 3.**
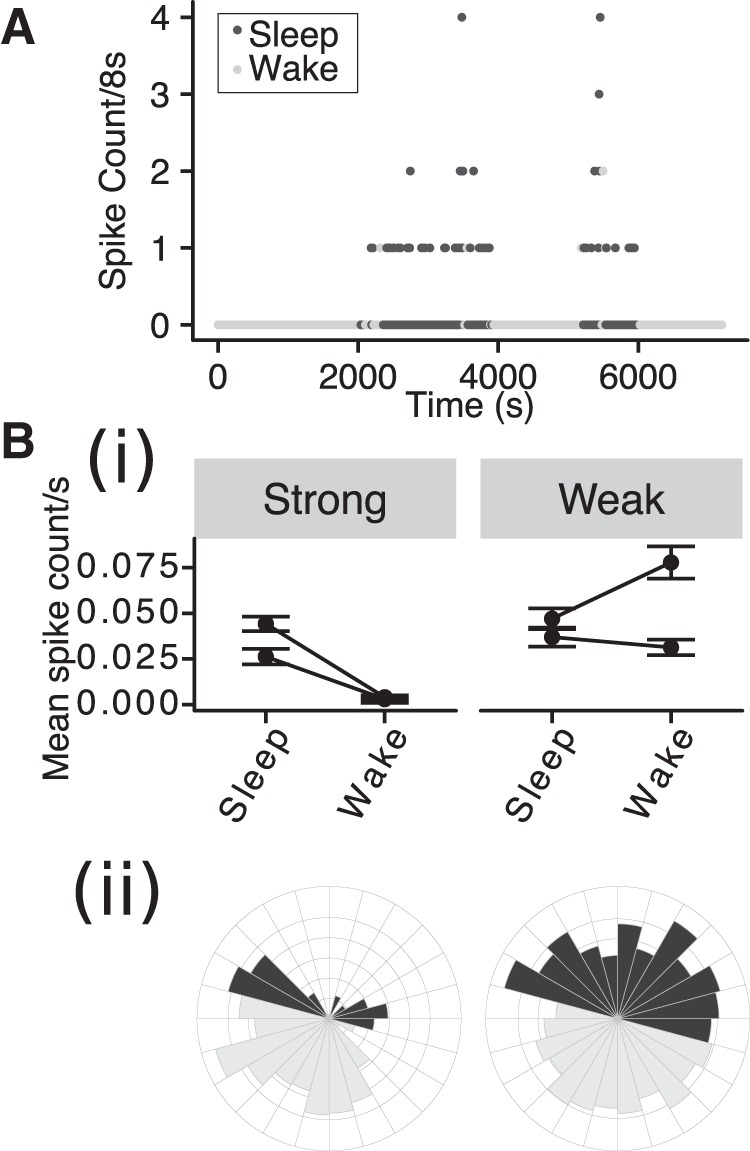
The probability of IIS is modulated by behavioral state in strongly phase-coupled animals. ***A***, IIS count/8-s interval versus time over 2 h of ECoG recording in a J20 mouse, with sleep and wake indicated by shading. ***Bi***, Mean spike rate in sleep and wake condition for strongly and weakly phase coupled animals. Error bars: 95% Confidence intervals (CI). ***Bii***, Circular histograms for a strongly (left) and weakly (right) phase coupled animals using conventions as in Figure 2*A*.

**Figure 4.**
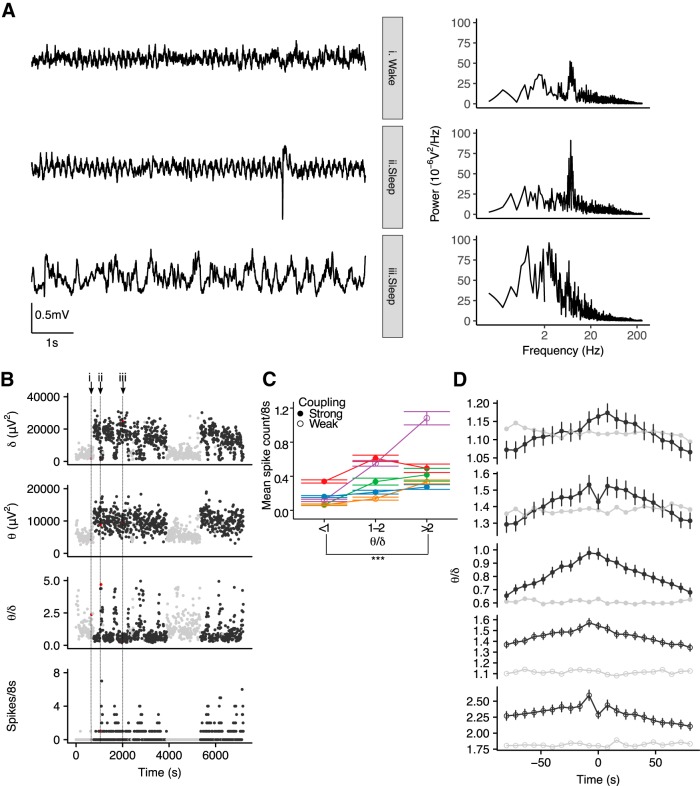
IIS occur during high θ/δ states. ***A***, 8-s ECoG signals (left) and corresponding power spectra (right) during different behavioral states recorded from a J20 mouse. A single IIS is seen in the sleep high θ state (ii). ***B***, Time series of δ power, θ power, θ/δ, and spike count per 8-s intervals across 2 h of ECoG recorded from the same J20 mouse as shown in ***A***. Black/gray symbols indicate sleep/wake as classified by simultaneous video data. Red symbols and vertical dotted lines indicate the 8-s intervals for which the ECoG signal is shown in ***A***. ***C***, Spike number per 8-s interval as a function of θ/δ in five animals (represented by different colors and connected by lines). The increase spike count in intervals with high θ/δ was seen in animals with both strong (filled symbols) and weak (open symbols) circadian phase coupling; ***p < 0.001. ***D***, IIS-triggered averages of θ/δ for five individual animals (black) and windowed averages triggered around 2000 randomly sampled points (gray) show an increased θ/δ around IIS. Strong/weak coupling shown in filled/open symbols. Error bars in ***B***, ***C*** represent 95% CI.

We observed that lossy and artifactual intervals resulted from movement and external sources of interference. We could not exclude the possibility that these events are nonrandomly distributed across the 24-h cycle. Nonrandom exclusion of intervals would impact the evaluation of coupling of IIS. We thus set a criterion: if >5% of all 8-s intervals were excluded due loss or artifact, the animal was excluded from calculations of coupling of IIS to circadian cycles, sleep-wake, and θ/δ. Two J20 animals which were included in Figure 1 were excluded from data reported in Figures 2–4 on this basis (25% and 16% of 8-s intervals excluded in these animals).

### Video analysis

Video data were manually scored to classify periods as “sleep” or “wake.” Based on previous reports, sustained inactivity ≥40 s was classified as sleep, while stationary periods <40 s and periods of movement were classified as wake (Pack et al., 2007). Postural shifts during sleep epochs did not break sleep epochs.

### Immunohistochemistry and imaging

Animals were killed by transcardial perfusion with *N*-methyl-D-glucamine (NMDG)-based saline solution (92 mM NMDG, 2.5 mM KCl, 1.25 mM NaH_2_PO_4_, 20 mM HEPES, 30 mM NaHCO_3_, 25 mM glucose, 10 mM MgCl_2_, 0.5 mM CaCl_2_, and sucrose to adjust osmolarity to 315–330 mOsm). Brains were postfixed with 4% paraformaldehyde for 24 h then washed and stored in PBS. Samples were put in 50% or 30% sucrose, PBS solution and 50% OCT solution for 24 h before cutting, then placed in the same solution and cut using a freezing microtome.

Fifty-micrometer sections were stored in PBS at 4°C. Slices were presoaked with 5% rabbit normal serum (RNS; Vector S-5000), 0.2% Triton X-100, PBS solution for 30 min at room temperature (RT), followed by incubation with 3% RNS, 0.2% Triton X-100, anti-choline acetyltransferase (ChAT; 1:500, Millipore #AB144P, RRID: AB_2079751), PBS solution for 48 h at 4°C. The sections were washed three times with PBS 0.2% Triton X-100 for 5min each and then incubated in 3% RNS, anti-goat biotinylated (1:200), DAPI (1:5000, Sigma D9542-1MG), PBS solution for 1 h at RT. After 3 PBS 0.2% Triton X-100 washings of 5min each, the sections were incubated with ABC reagent (Vectastain PK-6105 kit) prepared half an hour before using and stored in foil at 4°C containing 0.1% of A, 0.1% of B, 0.01% Triton X-100, PBS for 1 h at RT. After six PBS washings of 10 min each, the sections were put in three 3'-diaminobenzidine (Sigma D5905-50TAB), 0.02% CoCl_2_ (1% wt/vol), 0,04% (NH_4_)_2_Ni(SO_4_)_2_ (1% wt/vol) dH_2_0 solution for 30 min at 4°C over agitation. Then stained by adding 1.2% of fresh 1% H_2_0_2_ per slice for 10–20 s until the slice darkened. The slices were then transferred and washed in PBS, six times for 10 min each, mounted on a slide and dried for 30 min at 50°C then finally covered with Mowiol Embedding Medium and coverslips. Slides were stored at RT.

Imaging was performed on a Zeiss AX10 microscope using StereoInvestigator Software with a 5x/0.16 (420630-9900) apochromat air objective. Quantification was performed using StereoInvestigator Software “Optical Fractioner Workflow” probe with the following settings. Thickness of 50 µm was manually defined, and regions were selected using a 1.25x/0.03 (420310-9900) apochromat air objective for low magnification and then counted with a 10x/0.45 (420640-9900) apochromat air objective for high magnification. The border between medial septum (MS) and diagonal band of Broca (DB) was defined as a line between the two major island of Caleja. The regions were separated using different lines. The counting frame used was a square of 75-µm size and the grid was a square of 150-µm size. The counter was blind to genotype.

### Oral administration of Donepezil

Donepezil hydrochloride (Sigma Aldrich, D6821) was orally administered in a jelly. Mice were trained to voluntarily consume jelly following the protocol described by Zhang (2011). Mice were given placebo jelly or a jelly containing a Donepezil dose of 1.8 mg/kg. For experiments studying the effects of Donepezil on acetylcholinesterase (AChE) activity, jelly was given at 8 A.M. daily. For experiments studying the effects of Donepezil on IIS, jelly was given daily at either 8 A.M., or 8 P.M. to assess interactions of AChE modulation and circadian cycle. Since there was no effect of AChE on IIS, results were pooled.

### AChE assay

Quantitative measurements of AChE enzymatic activity were made using a modified Ellman method (Ellman et al., 1961; Rosenfeld et al., 2001). Stock solutions were acetylthiocholine iodide, used as the enzymatic substrate (ATH; 1.7 mg/ml in PBS, Sigma-Aldrich), 5,5’-dithio-bis(2-nitrobenzoic acid) (DTNB; 0.8 mg/ml in PBS, Sigma-Aldrich). Briefly, brains were rapidly dissected from either WT or J20 mice. Neocortex was isolated, weighed, and then homogenized using a Pellet Pestle (Sigma, Z 359971) in nine volumes of 0.1 M sodium phosphate buffer (pH 7.4; Patel et al., 2014). Five microliters of brain homogenate was aliquoted into each well of a 96-well plate, volume made up to 200 µl with PBS. DTNB (50 µl from stock) was added, followed by 50 µl of ATH substrate from stock. Measurement of absorption at 450 nm began immediately (<2 h from dissection) and was measured every 5 min for up to 30 min using a MRX microplate reader (Dynex Technologies). Thiocholine production in the test wells was expressed in units of nmol/min, calibrated with reference to the absorbance change over a range of concentrations giving a linear response using glutathione as the DTNB reactant (Eyer et al., 2003). Neostigmine (10 µM, Sigma-Aldrich) was used to completely inhibit AChE activity and establish that there was no baseline drift during the measurements.

### Human scalp EEG and foramen ovale (FO) electrode recordings

Human scalp EEG and FO electrode recordings were performed at the Massachusetts General Hospital, as described in detail previously (Lam et al., 2017). Scalp EEG electrodes were placed using the International 10-20 system, with additional T1 and T2 electrodes.

Sleep staging in patient data were performed by a board-certified clinical neurophysiologist (ADL) based on visual analysis of the full scalp EEG data. While dedicated electrooculogram and electromyogram channels were not recorded for these studies, the frontopolar scalp EEG electrodes allowed assessment of eye movements, while the frontopolar, frontal, and temporal electrodes allowed assessment of myogenic activity. Scalp EEG data were reviewed in 30-s epochs in the longitudinal anterior-posterior bipolar montage, using the Python module *wonambi* (https://github.com/wonambi-python/wonambi). Each 30-s epoch was classified as awake, non-REM (NREM)1, NREM2, NREM3, or REM, based on the American Academy for Sleep Medicine’s manual for sleep scoring.

Spike quantification in patient data were performed by a board-certified clinical neurophysiologist (ADL), using a custom-made GUI in MATLAB (Mathworks). The GUI displayed 15-s epochs of left and right sided FO data, in both bipolar and common reference montages (common reference = C2), along with the EKG trace to allow exclusion of EKG artifact. The reviewer could adjust amplitudes for each trace as needed. For the MCI patient analyzed, contact #3 from the left FO electrode did not record properly and was excluded from analysis. The reviewer marked all spikes in each epoch. Epochs were presented in consecutive order, but the reviewer was otherwise blinded to the sleep stage for each epoch during the review. Instantaneous spike rates were calculated by determining the total number of left FO and right FO spikes detected within all 30-s epochs of the recording (which corresponded to the sleep staging epochs above) and converting these rates to spikes per hour. Average spike rates within each sleep stage were calculated by summing the total number of spikes that occurred during each sleep stage and dividing by the total number of hours the patient spent in each respective sleep stage in the recording.

Spectral analysis of the FO electrodes was performed in MATLAB, using the freely available Chronux toolbox (Mitra and Bokil, 2007). Analysis was performed on the LFO1, LFO2, RFO1, and RFO2 channels, as these were the deepest contacts and thus least prone to noise or artifact. Channels were each normalized to zero-mean, unit-variance. Multi-taper spectrograms were calculated for each normalized channel, using the Chronux script *mtspecgramc* with the following parameters: frequency range: 1–20 Hz, window: 30 s; step size: 30 s; time-bandwidth product: 3, tapers: 5. This provided a spectral resolution of 0.2 Hz. An average spectrogram across all FO channels was then generated, and the average spectral powers within the δ-band (0–4 Hz) and θ-band (4–12 Hz) were then calculated.

### Statistics

Statistical data analysis was performed using R (version 3.2.0) including the “dplyr” (Wickham and Francois, 2015) and ggplot2 (Wickham, 2009) packages.

Assumptions for parametric tests were tested using Q-Q plots and residual plots. Data transformations or nonparametric tests were used for two-group comparisons in which test assumptions were violated.

For evaluating the effects of the fixed effects of age and genotype on the proportion of intervals containing more than one spike in APP^NL/F^ animals, the data first underwent a square-root transformation and then fit using a linear model:

$$\sqrt{Interval\,\;Proportion}∼Age+Genotype+\varepsilon$$

where ε is the error term.

The time of IIS was treated as circular variable. Each interval in which one or more IISs were detected was considered an event. The time of each event was evaluated as a phase of a circadian cycle. Circular data were analyzed using circular statistics by means of the “circular” package (Agostinelli and Lund, 2013). Circular outliers were identified using “CircOutlier” package (Rambli et al., 2016).

For tests entailing random variables, linear mixed models were fit using “lme4” (Bates et al., 2015). Significance was tested using a log-likelihood test comparing the full model to a null model without the factor of interest.

For evaluation of the relationship between spike count and θ/δ, we described each θ/δ value as a member of one of three levels: (1) θ/δ ≤ 1; (2) 1 < θ/δ ≤ 2, and (3) θ/δ > 2. We then modeled spike count (Poisson-distributed) as a function of levels of θ/δ, using the R package “MCMCglmm” (Hadfield, 2010). It should be noted that due to poor properties of a single model fitted across all animals (fitting animal as a random effect and θ/δ factor as a fixed effect), separate models were fitted to individual animals without including a random effect. Thus, the data do not allow for inference about the population.

Event-triggered averages of IISs were evaluated by considering each interval in which an IIS was detected as an event. If no intervals within ±80 s around the event were excluded, then the 160-s window was included in the calculation of the event-triggered averages, else the event was excluded from the averaging. An event-triggered average was also evaluated around 2000 randomly sampled points.

For comparing θ/δ in intervals with IIS to θ/δ in intervals preceding IIS, we considered only interval pairs where the preceding interval did not contain IIS and fit the model

$${\left(\theta /\delta \right)}^{1/4}∼Index+Subject+\varepsilon$$

where Index was a factor labeling whether the interval contained IIS or the preceding interval and modeled as a fixed effect, and Subject was a random effect with a random intercept.

For comparison of ChAT+ cells between genotypes, the model used was:

Estimated⁢ Count∼Genotype+Region+Subject+ε

where Genotype and Region were fixed effects and Subject was a random effect with a random intercept.

To study the effect of genotype and treatment of the Thiocholine production rate, the data of Thiocholine production was log-transformed. The model used was

log⁡(Thiocholine⁢ Rate)∼Genotype⁢ Treatment+Repeat⁢ ID+ε

where GenotypeTreatment was a fixed effect and RepeatID was a random effect with a random intercept. *Post hoc* tests for the linear model were performed using package “multcomp” with the Holm correction method (Hothorn et al., 2008). It should be noted that while the treatment levels of control and donepezil were independent, the neostigmine treatment was applied to a sample of WT control tissue and thus was not independent. This repeated factor was not accounted for in the model.

Significance was tested using α = 0.05. Two-sided hypothesis testing was used.

Superscripts following statistical reporting in the results section refer to the statistical table (Table 1).

**Table 1.** Statistical table

	**Data structure**	**Type of test**	**Confidence/Credible interval (CI) parameter**	**95% CI**
a	Normal (square root transformed)	*t* test	Difference of means of square root data	(0.20, 0.30)
b	Normal (square root transformed)	Linear mixed model	β-Genotype	(−0.01, 0.03)
			β-Age	(−0.02, −0.002)
c	IIS count data (analyzed with log-link function)	MCMC generalized model	Difference between estimates of θ/δ < 1 vs θ/δ > 2; provided for animals JF221, JF220, JF218, J0460, and J0456, respectively	(1.619, 2.122) (0.261, 0.471) (0.254, 0.478) (1.166, 1.392) (2.128, 2.372)
d	Normal (fourth root transformed)	Linear mixed model	β–Index	(−0.004, 0.008)
e	Normal	Linear mixed model	β-Genotype	(−1015.7, 1029.0)
f	Non-normal	Wilcoxon-signed rank test	Difference of medians	(0.08, 0.65)
g	Normal (log transformed)	Tukey contrasts	J20_Ctrl - WT_Ctrl	(−0.24, 0.03)
			WT_DPZ - WT_Ctrl	(−0.15, 0.12)
			J20_DPZ - WT_Ctrl	(−0.08, 0.19)
			WT_NSTG - WT_Ctrl	(−1.50, −1.23)
			WT_DPZ - J20_Ctrl	(−0.04, 0.23)
			J20_DPZ - J20_Ctrl	(0.02, 0.29)
			WT_NSTG - J20_Ctrl	(−1.40, −1.13)
			J20_DPZ - WT_DPZ	(−0.07, 0.21)
			WT_NSTG - WT_DPZ	(−1.49, −1.22)
			WT_NSTG - J20_DPZ	(−1.56, −1.29)
h	Normal	Paired *t* test	Difference of mean IIS rate	(−0.01, 0.03)

### Code and data accessibility

The processor script used for quantification of IIS, θ, and δ power in rodent ECoG data are available from http://www.opensourceinstruments.com/Electronics/A3018/HTML/SCPP4V1.tcl.

Code used for quantifying IIS in human data are available from https://github.com/mauriceaj/GUI-EEG_Spike_Annotation.

**Figure 5.**
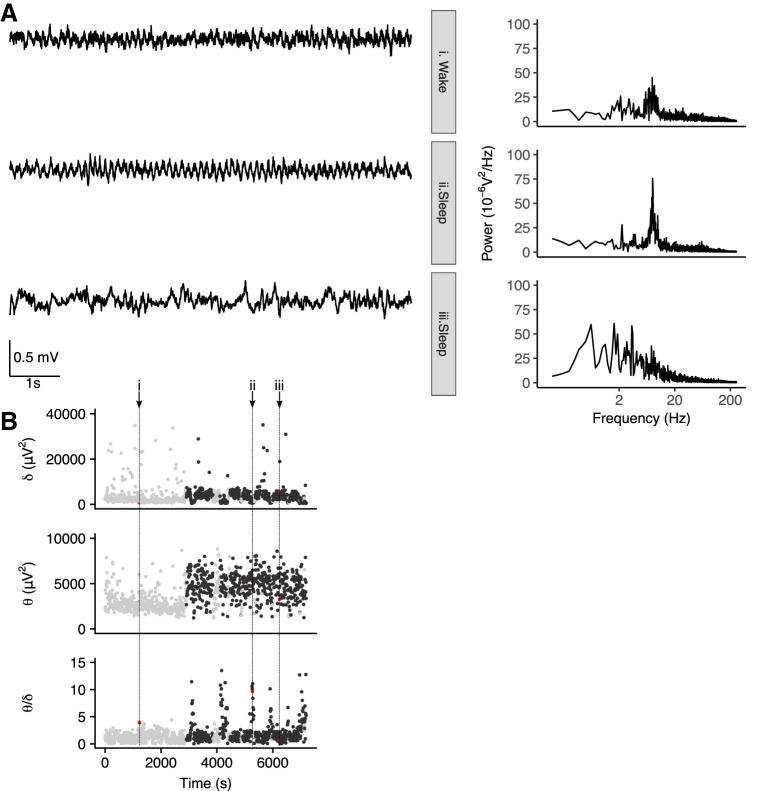
Transient increases in θ/δ are nonpathologic features of sleep. ***A***, 8-s ECoG signals (left) and corresponding power spectra (right) during different behavioral states recorded from a WT mouse. ***B***, Time series of δ power, θ power, and θ/δ per 8-s interval across 2 h of ECoG recorded from same WT mouse as shown in ***A***. Black/gray symbols indicate sleep/wake as classified by simultaneous video data. Red symbols and vertical dotted lines indicate the 8-s intervals for which the ECoG signal is shown in ***A***.

**Figure 6.**
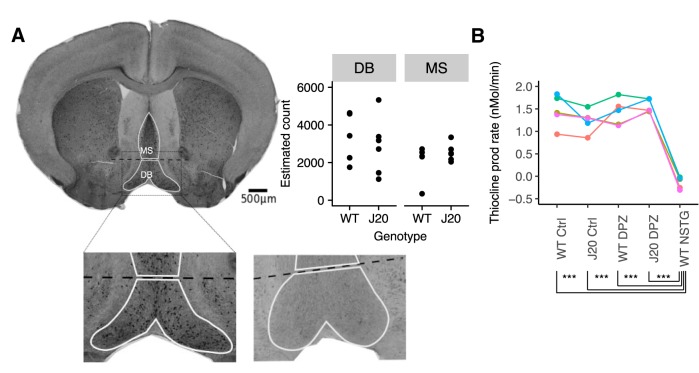
No evidence of cholinergic alterations in J20s. ***A***, Immunostained brain section showing ChAT+ cells in MS and DB. Lower panel shows zoomed in region of upper panel (left) and corresponding regions of a negative control stained section (right). Upper right: Quantification of stereological estimates of ChAT+ cell count in MS and DB in WT and J20. Points represent estimated counts in individual animals. ***B***, AChE activity was assayed by the rate of thiocholine production in brain homogenate from WT and J20 in control conditions and following oral administration of Donepezil (DPZ). The AChE activity was compared to a positive control of direct application of neostigmine (10 μM) to the brain homogenate. Experimental repeat groups are indicated by different colors and connected lines; ***p < 0.001.

The datasets used for Figures 1–6 (rodent data) are available from http://dx.doi.org/10.7488/ds/2319.

## Results

### Network hyperexcitability in mouse models of AD pathology

To establish circadian patterns of network hyperexcitability in J20 mice, we recorded ECoG activity from freely-moving J20 and littermate WT mice using wireless telemetry over a period of 3 d. As network excitability has been suggested to be an early event in AD pathogenesis (Vossel et al., 2013; Sarkis et al., 2016), we focused our study on ages which precede overt plaque pathology in J20s (Mucke et al., 2000).

As previously reported (Palop et al., 2007), nonseizure, IIS (Fig. 1*A*) were detected in J20 ECoG (note that while ictal activity was not assessed, we refer to these as interictal events due to the similarity with IIS that have been reported in the literature) . We applied automated event detection (see Materials and Methods), on 8-s intervals of continuous ECoG. The percentage of intervals in which 1 or more spikes were detected was negligible in WTs (mean percentage: 0.8%, SD = 0.7%, *n* = 8). In contrast, the percentage of intervals with 1 or more spikes was greater in J20s (mean percentage: 11.6%, SD = 5.1%, *n* = 18; *t*(23.98) = 10.6, *p* < 0.0001, *t* test on square root transformed data with Welch correction; Fig. 1*B*,*C*)^a^.

Seizures and IIS have been reported in numerous strains of transgenic mice that express hAPP and that exhibit Aβ pathology (Del Vecchio et al., 2004; Palop et al., 2007; Minkeviciene et al., 2009; Rasch and Born, 2013). However, it has been suggested that such network hyperexcitability is the result of overexpression of hAPP (Born et al., 2014). To determine whether network hyperexcitability is associated with Aβ pathology in the absence of hAPP overexpression, we performed telemetric ECoG recordings as above, in mice expressing the humanized Aβ sequence of APP (APP^NL/F^; Saito et al., 2014) and age-matched controls. We recorded from mice at ages preceding overt plaque pathology (eight months) and at ages where plaques begin to appear (12 months; Saito et al., 2014; Masuda et al., 2016). We found no significant effect of genotype in the proportion of intervals containing spikes between WT and APP^NL/F^ (*F*_(2,32)_ = 3.1, *R* ^2^ = 0.11, *p* = 0.06; Fig. 1*D*)^b^ with a negligible proportion of intervals with one or more spikes detected [mean percentage of intervals with one or more spikes, pooled across genotype and age = 1.2%, 95%CI (0.8%, 1.6%)]. A *post hoc* power calculation based on the effect size from the J20 group (Cohen’s *d* = 2.5) and the sample sizes of the APP^NL/F^ and WT groups yielded a power of >0.99 at α = 0.05 for an effect of genotype. Hence, we conclude that APP^NL/F^ mice show no evidence of network hyperexcitability compared to control animals.

### Circadian coupling of IIS

It has been suggested that seizure-related activity shows circadian fluctuations in epilepsies (Quigg, 2000). Hence, we next asked whether the likelihood of IIS in J20s varies across the day/night cycle. Quantifying the number of IIS per hour revealed that IIS are more frequent during daylight hours (inactive phase; Fig. 2*A*). We used circular statistics to extract measures of the phase coupling of IIS to the circadian cycle within individual J20 animals (see Materials and Methods). To evaluate the degree of phase coupling of IIS in each animal, we evaluated the mean angular vector length (ρ) from the time of IIS. ρ can vary between 0 (no phase coupling) and 1 (perfect phase coupling). To evaluate the time to which IISs were coupled, we extracted the mean coupling phase off IIS, expressed as a time on a 24-h cycle ($\varphi_{IIS}$).

The distribution of IIS phases differed significantly from a random distribution in all animals (Rayleigh test of uniformity: *p* < 10^−11^). The extent of phase coupling was variable across the sample of J20s (mean ρ_IIS_ = 0.24, SD = 0.13, *n* = 16; Fig. 2*B*).

Evaluating the coupling phase revealed that IIS occurred predominantly in the light condition (Fig. 2*A*). Across the sample of J20s, the mean $\varphi_{IIS}$ ($\overset{¯}{\varphi_{IIS}}$) confirmed this ($\overset{¯}{\varphi_{IIS}}$ = 15h05, ρ =0.38, *n* = 16, *p* < 0.0001, Rayleigh’s test; Fig. 2*B*). Inspection of the $\varphi_{IIS}$ distribution revealed potential outliers. Testing for outliers on a circular distribution (Rambli et al., 2016) identified four outliers. These four animals were among the five that showed a cluster of weakest phase coupling as measured by ρ_IIS_ (range: 0.06–0.11). We used the upper bound of the range of ρ_IIS_ of the four outlier animals to classify phase coupling as weak or strong. Henceforth, we refer to the five animals with ρ_IIS_ ≤ 0.11 as showing weak phase coupling, and the other 11 animals as showing strong phase coupling (ρ_IIS_ > 0.17).

### Sleep/wake modulation of IIS

Since IIS predominantly occurred in the normal inactive phase of the circadian cycle, we next asked whether this circadian modulation of IIS could be accounted for by the sleep/wake state of the animals. In a subset of J20s, we acquired simultaneous video recordings while recording ECoG data (*n* = 4). We manually scored the video and classified periods as sleep or wake (see Materials and Methods). Two of these four J20 animals showed strong circadian phase coupling of IIS, and two showed weak phase coupling. For the two animals that showed strong phase coupling of IIS, IIS occurred more frequently in sleep than during waking (Fig. 3*A*,*B*). In contrast, the modulation of IIS probability did not show a consistent pattern in animals showing weak phase coupling (Fig. 3*B*). This suggests that the strong phase coupling of IIS may be accounted for by differences in behavioral state across the circadian cycle.

### Brain state modulation of IIS in J20 mice

Sleep-related ictal and interictal activity is differentially modulated by REM and NREM sleep in different forms of epilepsy (Bazil and Walczak, 1997; Herman et al., 2001; Sedigh-Sarvestani et al., 2014; Ewell et al., 2015). REM and NREM can be distinguished by the relative power in the δ (defined here as 0.1–3.9 Hz) and θ (4–12 Hz) frequency bands, with high θ/δ associated with REM (Ewell et al., 2015) as well as waking exploration (Buzsáki, 2002). Thus, we next asked whether IIS are more likely to occur in particular brain states. To this end, we performed spectral analysis of the ECoG data from a subset of the mice (*n* = 5 J20s) in which a reference electrode was implanted at cerebellar coordinates (a noncortical reference for detection of cortical rhythms). ECoG recordings from J20 mice, exhibited periods showing a peak in θ-band power when animals were either awake (i.e., moving) or asleep, while periods of elevated δ-band power were seen during sleep (Fig. 4*A*). We evaluated the θ/δ ratio for each 8-s interval and related it to the number of IIS in the interval. Transient increases in θ/δ were observed during sleep and were associated with increased occurrences of IIS (Fig. 4*B*).

To quantify whether IIS were more likely in particular brain states, we next investigated the relationship between θ/δ and IIS count/8-s interval. As we were interested in discriminating between REM and NREM sleep, we limited the analysis to daylight hours when animals are more likely to be asleep. We used a value of θ/δ <1 and >2 to classify periods as NREM-like and REM-like, respectively (Ewell et al., 2015). This revealed significantly higher spike counts during REM-like versus NREM-like periods in all five animals (*p* < 0.0005 for all five animals, Markov Chain Monte Carlo generalized linear model; Fig. 4*B*)^c^. Interestingly, IISs were associated with increased θ/δ in animals showing both weak and strong phase coupling (Fig. 4*C*). Since sleep and wake are not predictive of IIS in animals with weak phase coupling, this suggests that there is a mismatch between θ/δ and behavioral state in animals with weak phase coupling. Moreover, high θ/δ states are predictive of IIS, regardless of behavioral state.

To examine the temporal dynamics of θ/δ around IIS, we evaluated the IIS-triggered average of θ/δ (Sedigh-Sarvestani et al., 2014) for 160-s window around each interval in which at least one IIS was identified. In all animals, θ/δ was increased around the time of IIS relative to θ/δ averaged around randomly sampled points (Fig. 4*D*). In three strongly phase-coupled animals, θ/δ returned to baseline levels within the 160-s window around the event. However, in the weakly phase coupled animals, θ/δ remained elevated above baseline levels in this window. The peak in the θ/δ IIS-triggered average did not occur at *t* = 0 in any of the animals. Since intervals neighbouring the IIS-containing interval show increased θ/δ, this suggests that the IIS contribution to spectral power did not underlie the association between increases in θ/δ and IIS probability. To further examine whether IIS could directly contribute to the increased θ/δ, we compared θ/δ in intervals with IIS to θ/δ in the preceding intervals only in cases where the preceding interval contained no IIS. We found no significant difference in θ/δ between intervals with IIS and the preceding interval (linear mixed model, χ^2^(1) = 0.35, *p* = 0.56; data not shown)^d^.

To determine whether the spectral ECoG patterns in J20 mice are a reflection of normal sleep or a result of pathology, we performed similar analysis of video-scored ECoG data from three WT mice. As in the J20, intervals of strong θ power were evident during wake and sleep, while periods of prominent δ–band activity were seen in sleep. Transient increases in θ/δ during sleep akin to those seen in J20s were also observed in all WT animals, suggesting that such increases are a feature of normal sleep, and not pathologic (Fig. 5). To compare the distribution of θ/δ during sleep between genotypes, we calculated the range and 90th percentile of θ/δ while animals were asleep (using data for which we had video scoring). Group sizes were too small for statistical comparison but suggested that θ/δ values spanned a narrower range in J20 mice than in WT mice [J20 mean range = (0.02, 10.0), 90th percentile = 2.4, SD(1.1), *n* = 4; WT mean range = (0.04, 19.3), 90th percentile = 5.4, SD(1.4), *n* = 3; data not shown].

### No evidence of cholinergic changes in J20 mice

Cholinergic levels exhibit a circadian modulation (Hut and Van der Zee, 2011), and high cholinergic tone is implicated in generating θ oscillatory states (Buzsáki, 2002). In addition, cholinergic dysfunction has been suggested to be a key feature of AD pathogenesis (Craig et al., 2011). Recently, it has been suggested that cholinergic alterations may contribute to network excitability in the Tg2576 model of AD (Kam et al., 2016). Hence, we hypothesized that cholinergic changes might underlie the brain-state dependent modulation of IIS in the J20 mice. We used immunohistochemistry to quantify the number of ChAT+ cells in the MS and DB and asked whether the number of ChAT+ cells differs between J20 (*n* = 7) and WT (*n* = 5) mice. Fitting a linear mixed model to the data, we found no effect of genotype on the estimated number of ChAT+ cells in the MS or DB (linear mixed model, χ^2^(1) = 0.0002, *p* = 0.99; Fig. 6*A*)^e^.

AChE activity is reduced in AD (García-Ayllón et al., 2011). We assayed cholinergic function by measuring AChE activity. AChE activity was quantified by estimating the rate of thiocholine production in neocortical brain homogenates (see Materials and Methods). There was no significant difference in the rate of thiocholine production in brain homogenates prepared from WT and J20 mice (V = 15, *p* = 0.06, *n* = 5 WT/J20, Wilcoxon signed rank test, matched by day of assay; Fig. 6*B*)^f^. We also wanted to directly test the effect of modulation of ACh levels on IIS. However, using oral administration of Donepezil at a dose previously suggested to achieve clinically relevant drug plasma levels (Dong et al., 2009) was ineffective at altering AChE activity in brain homogenates. In contrast, a positive control treatment of direct application of neostigmine to brain homogenate led to a significant reduction in AChE activity (linear mixed model: χ^2^(4) = 73.5, *p* < 0.0001; *post hoc* using Tukey paired comparisons: *p* < 0.0001 for neostigmine versus each of the treatment and genotypes; *p* > 0.05 for all other group comparisons; Fig. 6*B*)^g^. Two days of oral Donepezil administration at this dose did not affect the IIS rate in J20 mice (*t*_(11)_ = 0.8, *p* = 0.43, paired *t* test; data not shown)^h^.

### Sleep stage modulation of IIS in human AD

The first intracranial recordings in humans with AD were recently reported and demonstrated marked activation of mesial temporal lobe (mTL) IIS during sleep compared to the awake state (Lam et al., 2017). We further analyzed the combined scalp EEG and intracranial electrode recordings from these two patients to better understand the relationship between sleep stage and mTL IIS rate in AD patients. One patient with advanced AD did not achieve REM sleep but showed mTL IIS preferentially during NREM sleep as opposed to waking states (Table 2, patient 1). The second patient was a 67-year-old woman with amnestic MCI (aMCI), an early stage of AD that is thought to correspond to the early stage of AD modeled in our young J20 mice. The data from this patient were used to compare the frequency of IIS in wake, NREM, and REM states.

**Table 2.** Average mTL spike rates were evaluated from FO electrodes and related to sleep stage as assayed by scalp EEG in two patients with AD

	**Patient 1 (AD dementia)**		**Patient 2 (aMCI)**	
**Sleep stage**	**Total hours in record**	**Average spike rate (spikes/hour)**	**Total hours in record**	**Average spike rate (spikes/hour)**
Wake	4.7	11	5.2	329
NREM1	0.7	31	1.5	670
NREM2	2.1	80	3.8	739
NREM3	1.4	62	3.1	903
REM	0	n/a	0.7	159

We analyzed 14.25 consecutive hours of combined scalp EEG and FO recordings from the aMCI patient, which spanned from ∼7 P.M. on the first day of FO recording (FOD1) to 9:15 A.M. the following morning (FOD2). Further recordings were not analyzed, as the patient was initiated on treatment with the anticonvulsant levetiracetam on the afternoon on FOD2. Of note, the patient underwent implantation with FO electrodes on FOD1 from ∼12:40 to 1:50 P.M. and received sevoflurane, Propofol, and midazolam during the procedure. She was awake and answering questions appropriately by 2:15 P.M. on FOD1.

**Figure 7.**
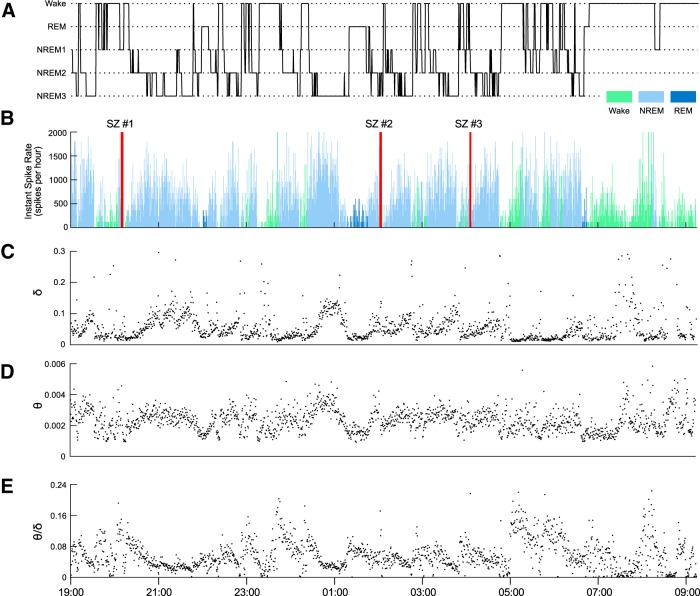
Sleep stage coupling of mTL spiking in a human with aMCI, a suspected early stage of AD. ***A***, Hypnogram showing the patient’s sleep architecture, spanning from ∼7 P.M. on FOD1 to 9:15 A.M. on FOD2. ***B***, Bar plot showing instantaneous mTL lobe spike rates over the course of the recording. Bars are colored by sleep stage, with light green for Wake, light blue for NREM (includes NREM1, NREM2, and NREM3), and dark blue for REM. The patient had three brief subclinical seizures (SZ) from the left FO electrodes during this recording, the timing of which is depicted by red vertical bars. ***C–E***, Plots showing (***C***) δ power (0–4 Hz), (***D***) θ power (4–12 Hz), and (***E***) θ/δ ratio of bilateral mTL activity, based on FO electrodes recordings. Dots represent the spectral power for each nonoverlapping 30-s window of the recording. Power is measured in arbitrary units.

We performed sleep staging of the recording using the full scalp EEG data and measured mTL spike rates using the bilateral FO electrode data (Fig. 7*A*,*B*). As described previously, we found that mTL spiking in the aMCI patient was largely activated during sleep. In contrast to what we found in the young J20 mice, mTL spiking in the aMCI patient occurred with highest frequency during NREM sleep stages, particularly during NREM3, and were lowest during REM sleep (Fig. 7; Table 2). mTL IIS rates during REM sleep were also markedly lower than during wakefulness (Table 2). We also calculated spectral power in the θ- and δ-bands, as well as the θ/δ ratio, in the FO electrodes across sleep states (Fig. 7*C–E*). Increases in both θ and δ power were seen with deepening stages of NREM sleep, while a reduction was seen with REM sleep. In contrast to what we observed in the J20 mice, the θ/δ ratio was reduced during periods of highest spike frequency (Fig. 7*E*).

## Discussion

Network hyperexcitability is a feature of AD. Here, we compared patterns of network hyperexcitability in two rodent models of AD, as well as in two AD patients, to reveal shared phenomenological features with the disease. We show that while J20 (hAPP overexpressing) mice exhibit frequent IIS as previously reported, APP^NL/F^ mice (which express APP at physiologic levels) do not show evidence of network hyperexcitability. Moreover, IIS in J20s occur primarily during daylight hours, and this circadian fluctuation is accounted for by an increased probability of IIS during sleep. Interestingly, we found that IIS in J20 mice are modulated by brain state, with increased likelihood of IIS in brain states with high θ/δ activity, a marker of REM sleep. In contrast, patients with AD showed prevalent IIS during NREM sleep. Moreover, in the one AD patient who exhibited REM sleep, IIS frequency was lowest in REM compared to other states.

### Circadian dysfunction and network hyperexcitability in AD

Brain network hyperexcitability in the form of IIS and seizures has now been reported in numerous models of AD pathology (for review, see Scharfman, 2012; Born, 2015). Our data, along with those reported by others (Born et al., 2014; Kam et al., 2016) reveal that network hyperexcitability in animals models of AD can be modulated by the circadian cycle. Circadian disturbances in AD include sleep fragmentation, increased daytime somnolence, and sundowning, the phenomenon in which neuropsychiatric symptoms are heightened late in the day (Peter-Derex et al., 2015). Animal models of AD have also been reported to show disturbances in the circadian cycle, some of which overlap with patterns of circadian alterations seen in patients (Huitrón-Reséndiz et al., 2002; Vloeberghs et al., 2004; Wisor et al., 2005; Jyoti et al., 2010; Sterniczuk et al., 2010; Duncan et al., 2012; Roh et al., 2012). Our findings of circadian modulation of network hyperexcitability in AD raise the question of whether IIS might causally contribute to the alterations in circadian-coupled behavior observed in AD. Future work investigating the effects of anti-epileptic drugs on circadian alterations in AD would go toward answering this.

### Brain state modulation of network excitability

Here, we report that IIS in J20 animals are modulated by θ/δ, with higher IIS rates seen in states of high θ/δ during sleep. The spectral patterns of ECoG that we report here are in line with previous reports in WT mice, that have shown increases in cortical EEG θ power in REM sleep relative to wake and NREM (Brankack et al., 2010). We also report transient increases in θ/δ in sleep in both WT and J20 mice. Since these increases in θ/δ occur in both WT and J20s, they are likely to be indicative of REM sleep periods (Ewell et al., 2015). Given that J20 animals with strong circadian phase coupling show highest IIS rates during sleep this suggests that IIS in these animals are associated with REM sleep.

An alternative explanation for the association between IIS and high θ/δ during sleep may be that IIS occur during ectopic θ in sleep, in the absence of a concomitant drop in muscle tonus. A phenomenon of ictal activity during ectopic θ has been reported in a mouse model of Huntington’s disease (Pignatelli et al., 2012). Without simultaneous EMG recordings, the present data cannot conclusively distinguish between REM states and ectopic θ. In the human data, analysis of θ/δ ratios showed that these were lowest during periods of highest IIS frequency. This argues against the idea of IIS coupled to ectopic θ in humans, although a more definitive assessment will require data from more AD subjects as well as healthy elderly controls.

Our finding of an association between IIS and high θ/δ is in line with recent reports that young Tg2576 model of AD as well as mice overexpressing WT-hAPP also demonstrate IIS predominantly during states of high θ which the authors suggest is indicative of REM sleep (Kam et al., 2016).

The findings that IIS in multiple mouse models of AD are most likely to occur in REM-like states begs the question of what makes REM a proictal state in these models. Both REM sleep and the awake state share common features of high θ/δ activity and high cholinergic tone (Vazquez and Baghdoyan, 2001; Lee et al., 2005), yet IIS occur much less frequently in the awake state in these models. There are several potential explanations for this. Firing rates of hippocampal neurons increase during REM (Grosmark et al., 2012), which might contribute to the propensity to seize. In addition, systems that normally show distinct activity in REM sleep versus waking and NREM sleep might contribute to the proictal REM state in these models (Sedigh-Sarvestani et al., 2014; Ewell et al., 2015; Kam et al., 2016). Unlike cholinergic neurons, which increase their activity in both REM and waking, monoaminergic neurons in brainstem nuclei (including the locus coeruleus and the tuberomammillary nucleus) as well as the dorsal raphe nucleus of the hypothalamus, show differential activity between these brain states. These neurons are highly active in waking, exhibit low firing rates in NREM sleep, and are quiescent during REM sleep (Lee and Dan, 2012). It may be that brain state modulation of one or more of these systems is disrupted in these mouse AD models, and other forms of epilepsy which show REM coupling (Sedigh-Sarvestani et al., 2014; Ewell et al., 2015).

The present study quantified cholinergic neurons in MS and DB. Cholinergic neurons in laterodorsal tegmental and pedunculopontine tegmental nuclei of the pontomesencephalic tegmentum have been suggested to control REM onset (Van Dort et al., 2015). In the rat, these neurons have been shown to be active during both wake and REM; however, firing rates are higher in REM, and correlate with θ/δ (Boucetta et al., 2014). Thus, changes to these neurons are also potential candidates for mediating the proictal nature of REM sleep in J20 mice.

Kam et al. (2016) reported that MS-DB cholinergic neuron number was unchanged in young Tg2576 mice. However, they found evidence to support the notion that overactivity of cholinergic neurons might contribute to IIS by showing that antagonism of muscarinic receptors reduced IIS in these animals. Hence, they concluded that IIS during REM might be the result of cholinergic hyperfunction. We did not find evidence for cholinergic changes in J20 mice as quantified by the number of cholinergic neurons in MS-DB, or AChE activity. If cholinergic activity is indeed unaltered in J20 mice, future experiments using muscarinic antagonism in J20 mice could be used to investigate whether atropine can act to reduce IIS by reducing overall neuronal excitability, rather than by reversing cholinergic hyperfunction.

Our assay of cholinergic function was based on measurements of AChE enzymatic activity in brain homogenate. There was no significant difference between AChE levels in WT and J20, or with Donepezil treatment. While it is possible that postmortem degradation of AChE could have masked differences in AChE levels, the robust effect of neostigmine supports the conclusion that the tissue contained functional AChEs.

In a subset of our animals, IIS were weakly coupled to the circadian cycle and the sleep-wake pattern but were still modulated by θ/δ. This suggests that the relationship between θ/δ and behavioral state might be disturbed in these animals. It is possible that these animals also exhibited greater disturbances in other elements of the circadian cycle, such as a circadian decoupling of sleep quantity/quality.

During both REM and NREM, hippocampal neurons have been shown to replay firing patterns that were experienced before sleep (Skaggs and McNaughton, 1996; Louie and Wilson, 2001), and such precisely timed sequences are likely to be involved in the memory facilitation role of sleep. IIS are thought to arise from depolarization and synchronous firing of neurons. This firing is followed by an inhibition and reduction of firing (Holmes and Lenck-Santini, 2006). Thus, IIS during sleep are likely to interfere with the coordinated replay of firing sequences, and consequently, would be expected to contribute to memory impairments. In support of this, it has recently been shown that reducing IIS by treatment with anti-epileptic drugs, rescues memory deficits in J20s (Sanchez et al., 2012).

### Relationship between IIS and AD pathology in mouse models

Here we report that while IIS are prevalent in hAPP overexpressing mice, APP^NL/F^ mice that exhibit Aβ pathology without APP overexpression, do not exhibit IIS at two ages preceding widespread plaque deposition (eight and 12 months). This finding is in line with other reports that it is overexpression of hAPP that is causal in generating network hyperexcitability in these animal models (Born et al., 2014; Xu et al., 2015; Kam et al., 2016). An alternative explanation of the presence of IIS in J20 but not APP^NL/F^ mice may be differences in the levels of Aβ between the two models. However, levels of soluble Aβ in six-month-old J20 and 12-month-old APP^NL/F^ are comparable, and levels of total Αβ are higher in APP^NL/F^ (Shankar et al., 2009; Saito et al., 2014). Thus, it is unlikely that higher levels of Aβ in the J20s are a cause of IIS in this model.

Interestingly, APP^NL/F^ mice begin to exhibit cognitive deficits at eight months of age (Masuda et al., 2016), which suggests that cognitive deficits at these ages are not the result of IIS, as has been suggested for J20s (Sanchez et al., 2012). Moreover, differences in the types of memory affected in J20 and APP^NL/F^ at ages preceding overt plaque deposition have been reported. Specifically, four- to six-month-old J20s show impairments in hippocampal dependent spatial memory (Sanchez et al., 2012). In contrast, in eight-month-old APP^NL/F^ mice, spatial memory as assayed by a place preference task is intact. However, place-avoidance memory, which is also dependent on amygdala circuits (Wilensky et al., 2000), is impaired (Masuda et al., 2016). It may be that hippocampus dependent processes are susceptible to interference by IIS while the disturbances in the nonhippocampal circuits result from processes independent of IIS.

### Differential sleep-stage coupling between mouse models of AD and human AD

Lam et al. (2017) recently used intracranial electrode recordings to detect mTL IIS in two AD patients without a history of epilepsy. Here, we report that in these patients, IISs were predominantly associated with NREM sleep (i.e., low θ/δ). In the patient with aMCI, IIS occurred most frequently in NREM3 sleep and were least frequent in REM, with a >4.5-fold difference in spike rates between NREM3 and REM. In the AD patient, frequent IIS were seen during NREM sleep, although REM sleep was absent from this patient’s brief recording, in line with previous reports of REM deficits in AD (Vitiello et al., 1984). Our findings from intracranial electrodes in AD patients are consistent with prior scalp EEG studies by Vossel et al. (2016), who reported that epileptiform discharges are highly prevalent in sleep stages >2 (although the authors did not differentiate between REM and NREM sleep). Although the means of characterising sleep differed between rodents and patients, combined, these results point to important differences in sleep stage coupling of epileptiform activity between rodent AD models and humans with AD and suggest that the specific mechanisms that underlie hyperexcitability in AD may differ between certain mouse models and humans.

Analysis of ictal and interictal activity in epilepsy patients has led the view that NREM sleep is a generally proictal state, whereas REM sleep is an anti-ictal state (Sammaritano et al., 1991; Herman et al., 2001; Minecan et al., 2002; Ng and Pavlova, 2013). Many animal models of epilepsy have also shown that seizures are more frequent in NREM and rarely occur in REM (Shouse et al., 2000). Interestingly, rodent models of the same type of epilepsy can still exhibit differences in the sleep-stage coupling of epileptiform activity. For example, in both the kindling as well as the pilocarpine models of temporal lobe epilepsy in rats, IIS are most common during NREM sleep (Colom et al., 2006; Gelinas et al., 2016). In contrast, rats with either the tetanus toxin or the low-dose kainate models of temporal lobe epilepsy have seizures that occur most commonly during REM sleep (Sedigh-Sarvestani et al., 2014; Ewell et al., 2015). Based on this, we hypothesize that different mouse models of AD may have specific mechanisms underlying their network hyperexcitability, which could be differentially expressed through sleep-stage coupling of IIS. We propose that sleep-stage coupling of IIS should be an important factor for identifying mouse AD models that more closely resemble the EEG signature of network hyperexcitability in human AD.

## Synthesis

Reviewing Editor: William Stacey, University of Michigan Decisions are customarily a result of the Reviewing Editor and the peer reviewers coming together and discussing their recommendations until a consensus is reached. When revisions are invited, a fact-based synthesis statement explaining their decision and outlining what is needed to prepare a revision will be listed below. The following reviewer(s) agreed to reveal their identity: Laura Ewell, Paula R. Haynes

We have discussed the paper and agree that the findings are very interesting. There are several concerns addressed below that need to be addressed before the paper can be accepted for publication. As you will see from this consensus, the two reviewers were very much in agreement, even before the discussion began.

1. The applicability of these results to human disease is overstated and not well substantiated. Specifically, there is only one patient with REM sleep, and this is insufficient to make the claims regarding the difference between NREM and REM sleep in humans. This was summed up by R2 “With only one patient achieving REM sleep, it is not possible to make conclusions at this level of detail. I think it is OK to report the data in Figure 6, just be more conservative with the implications. For example discussion sentence starting at line 541 ‘In contrast, patients with AD showed the highest IIS rates during NREM sleep, and were least likely to have IIS during REM’. Please tone this down, and at the very least don't refer to patients, as only one patient was studied in respect to REM/NREM.” And R3 added “another study was cited as evidence of coupling between IIS in patients and NREM sleep (Vossel et al 2016.) However, this study indicates only a high prevalence of epileptiform activity during sleep stages 2 or greater. It is not clear if this includes or does not include REM.”

2. Another concern is the comparison between human data, which has official sleep staging that includes EMG, and mice that have only the theta/delta ratios calculated. This is very difficult to reconcile, especially since there is no validation for the mice. It is possible that states defined as REM solely based on theta/delta ratio may not actually reflect REM sleep, but rather a disease-specific brain state occurring during SWS and coupled to IIS. In agreement with this, IIS were coupled to ectopic theta and reduced delta activity during SWS in a Huntington's mouse model (Neurobiol Dis. 2012 Dec;48(3):409-17). Another mouse AD model was cited which also exhibited REM-coupled IIS (Kam et al 2016), but again REM was defined chiefly by theta/delta ratio without EMG/EOG. Differences and caveats in analysis methods for humans vs. mice should be discussed and conclusions should be updated to include this possibility. (Do the human patients in the current manuscript also have IIS coupled to NREM invaded by ectopic theta?)

3. Similarly, the method of analyzing the theta/delta ratios in ECoG is hard to compare with LFP, where it is typically used. It is unclear whether the signals (especially theta) are clear/ robust in the ECog recording. In particular, the authors observe stronger power in the theta band during sleep compared to wakefulness (Figure 4). This is unexpected and makes this reviewer question the quality of the signals. For this concern, the reviewers suggest showing raw signals to assure the data are valid. I suggest you try this, and hopefully the effect can be seen. Their suggestion is to show representative J20 and control ECOG traces during NREM, REM and wake states with the same power analysis repeated during an active wake state to verify the quality of trace (the expectation is that there would be a robust theta signal when the animal is awake and moving). Ideally, you could verify REM with EMG, but that may not be available. (EMG would be expected decrease during SWS vs. wake and decrease even further during REM). If these raw traces are not available, or are not helpful in validation, you should find another way to validate this finding-in other words, it is not absolutely necessary to show raw signals (which are the way the reviewers feel would best validate the findings), but it is necessary to show something that validates the method and results.

4. Discuss/ investigate the possibility of ectopic theta occuring during SWS as a confound to their definition of REM (and reanalyze human patient data for evidence of ectopic theta if possible.) It would also be helpful to look at the total amount and bout duration of REM vs NREM episodes in J20 vs. controls to get a better idea of whether REM in J20s is normal (they should already have all of this data.)

5. Add to discussion the possibility that J20- IIS related memory deficits may be hippocampal, whereas NL/F may be non-hippocampal

6. any software that was used should be made available, in line with our new ‘Policy on Computer Code and Software’ outlined in http://www.eneuro.org/content/general-information#policies

The following minor concerns should also be addressed:

1.) Please indicate if the mice used for experiments in figures 3 and 4 are the same or different than those used in figures 1 and 2? If they are the same, is strong vs. weak phase coupling maintained within each animal across experiments?

2.) The use of APP-NL/F animals that were only backcrossed to B6 3x rather than the standard 6x is not optimal especially given that littermates were not used as controls. However these animals were only used in Figure 1 and do not compromise the major conclusions of the manuscript.

3.) Please give more detail on the method used to prepare brain homogenate for AChE activity. Is it possible that homogenate preparation results in some degradation of AChE and obscures differences between groups?

4.) Some places in the text say ‘thiocline’ instead of thiocholine, this is confusing and should be fixed.

5.) Cholinergic neurons in MS and DB were analyzed, but not PPT and LDT. PPT/LDT neurons also promote REM and could be a source of cholinergic deficits.

6.) Inclusion of some statistics in figures and others in the statistics table is confusing. It would help to make things consistent and/or cite the statistics table in figure legends and text where it is relevant.

7.) Clarification of the term interictal spike vis-a-vis animals that do not have seizures, comment 5 of R2.

8.) Clarify the term ‘lossy’ in line 170

The comments below are those of the two reviewers. The Synthesis above represents the final decision the three of us made; the comments below are for reference and further explanation.

Reviewer 1 withdrew and did not submit comments

Reviewer 2: The paper is well written. The authors clearly describe their questions and data. They find interesting differences between AD models with respect to hyperexcitability, and find that hypersynchronous discharges are more prominent during sleep. The reviewer has a few suggestions to making the paper rhetorically stronger, and also raises one major concern

about the sleep analysis to distinguish REM from NREM.

(1)The authors find a big differences between the overexpression model of APP (J20) and the human mutation model (APPNL/F ). The manuscript would be rhetorically stronger if this difference was brought forward - in the introduction and discussion. Can the authors speculate why the overexpression model might cause hyper-synchronous discharges?

(2)My major concern for the study is the ability of the authors to properly separate REM and NREM sleep. They are using voltage oscillation criteria that typically are used on hippocampal LFP. It is unclear whether the signals (especially theta) are clear/ robust in the ECog recording. In particular, the authors observe stronger power in the theta band during sleep compared to wakefulness (Figure 4). This is unexpected and makes this reviewer question the quality of the signals. I would ask that raw signals are shown. Furthermore, the same power analysis should be done on a subset of the data when the animals are active (during the dark part of the sleep/wake cycle). Specifically, when animals have been verified to be moving with video monitoring. In this case, the expectation is that there would be a robust theta signal. Without seeing these data, it is difficult to assess whether the theta/delta ratio being used is properly distinguishing between REM/ NREM.

(3)For the REM/ NREM modulation of IIS's of the 5 animals used, please clarify in the text whether they had strong or weak sleep/wake cycle modulation.

(4)The human data points toward spikes occurring during sleep, but please take out conclusions about REM/NREM modulation. With only one patient achieving REM sleep, it is not possible to make conclusions at this level of detail. I think it is OK to report the data in Figure 6, just be more conservative with the implications. For example discussion sentence starting at line 541 “In contrast, patients with AD showed the highest IIS rates during NREM sleep, and were least likely to have IIS during REM”. Please tone this down, and at the very least don't refer to patients, as only one patient was studied in respect to REM/NREM.

(5)I understand that the field uses the term ‘inter-ictal spike’, and that is why it is used here. I think it is problematic when there are no ictal events (seizures) recorded, so the term inter-ictal is misleading. Perhaps when the term is first used, state that the reason it is used is because the observed activity resembles inter-ictal spikes that occur in networks that generate ictal discharges and be very clear that there are no ictal events observed in the J20 animals. Along these lines, please be very careful with terms. In the discussion, instead of asking why REM is ‘pro-ictal’ in the J20 model, ask instead why REM is a ‘hyperexcitable’ state. Pro-ictal literally means promoting seizures when instead what you observe is that it may promote spikes (or hypersynchronous discharges).

(6)Line 170: Please clarify the term ‘lossy’

Reviewer 3:

Major findings:

The authors compare two mouse models of Alzheimer's disease to determine the suitability of each for modeling neuronal hyperexcitability. ECoG recordings as well as video analysis of behavioral state are used to quantify epileptiform activity in conjunction with sleep/wake states. Results show that mice overexpressing a humanized mutant APP in neurons (J20) exhibit frequent interictal spikes (IIS), whereas animals expressing mutant APP at physiological levels do not exhibit IIS. Further characterization of IIS in J20 mice shows that the majority of this activity occurs during sleep, as in human patients. However, reanalysis of sleep-stage data from two previously published human patients indicates a high prevalence of IIS during NREM sleep, whereas mouse data presented seems to indicate that IIS occur primarily during REM sleep. These results suggest the more physiological NL/F mouse model cannot be used to study hyperexcitability and that caution should be used in generalizing hyperexcitability phenotypes from mouse models to humans since they may have different underlying mechanisms.

Major concerns:

A key conclusion of this manuscript is that coupling of IIS to sleep stage is different between human patients (IIS coupled to NREM sleep) and a mouse model of AD (IIS coupled to REM sleep) and this brings into question the relevance of mouse models to the human disease state. There are a couple of issues with these conclusions and the manuscript should be updated to reflect these issues:

1.) The methods used to distinguish NREM from REM sleep are different in mouse and human experiments and these differences may affect conclusions. While only the theta/delta ratio was used to determine REM in mice, analysis of human EEG traces was scored manually following AASM guidelines, which take into account additional measurements such as EOG and EMG to define REM vs NREM. Without EOG/EMG data in the mouse model, it is possible that states defined as REM solely based on theta/delta ratio may not actually reflect REM sleep, but rather a disease-specific brain state occurring during SWS and coupled to IIS. In agreement with this, IIS were coupled to ectopic theta and reduced delta activity during SWS in a Huntington's mouse model (Neurobiol Dis. 2012 Dec;48(3):409-17). Another mouse AD model was cited which also exhibited REM-coupled IIS (Kam et al 2016), but again REM was defined chiefly by theta/delta ratio without EMG/EOG. Differences and caveats in analysis methods for humans vs. mice should be discussed and conclusions should be updated to include this possibility. (Do the human patients in the current manuscript also have IIS coupled to NREM invaded by ectopic theta?)

2.) Broader conclusions about sleep-stage coupling of IIS in humans- specifically a lack of IIS during REM- may be a bit premature given the data shown here and cited. Only a single human patient with both REM and NREM sleep stages was analyzed in this study for segregation of IIS to brain state. Although a second patient was studied, this patient lacked the REM state entirely, suggesting AD-REM deficits, but should not be used for conclusions about a lack of IIS during REM in humans. Last, another study was cited as evidence of coupling between IIS in patients and NREM sleep (Vossel et al 2016.) However, this study indicates only a high prevalence of epileptiform activity during sleep stages 2 or greater. It is not clear if this includes or does not include REM.

Minor concerns:

1.) Please indicate if the mice used for experiments in figures 3 and 4 are the same or different than those used in figures 1 and 2? If they are the same, is strong vs. weak phase coupling maintained within each animal across experiments?

2.) The use of APP-NL/F animals that were only backcrossed to B6 3x rather than the standard 6x is not optimal especially given that littermates were not used as controls. However these animals were only used in Figure 1 and do not compromise the major conclusions of the manuscript.

3.) Please give more detail on the method used to prepare brain homogenate for AChE activity. Is it possible that homogenate preparation results in some degradation of AChE and obscures differences between groups?

4.) Some places in the text say ‘thiocline’ instead of thiocholine, this is confusing and should be fixed.

5.) Cholinergic neurons in MS and DB were analyzed, but not PPT and LDT. PPT/LDT neurons also promote REM and could be a source of cholinergic deficits.

6.) Inclusion of some statistics in figures and others in the statistics table is confusing. It would help to make things consistent and/or cite the statistics table in figure legends and text where it is relevant.

7.) Regarding the relationship between IIS and memory deficits noted in the discussion, is it possible that IIS, as in J20 mice, specifically disrupt hippocampal-dependent memory, whereas cognitive deficits in APP-NL/F animals are the result of non-hippocampal disruption? For example, NL/F mice are normal in a place preference task, but abnormal in a place avoidance task. Both of these should involve the hippocampus, but only the second case may require the amygdala for fear/avoidance, suggesting possible deficits in amygdala, but not hippocampus.
